# Supplementary material for: Are carbon emissions trading and green financial instruments synergistic? -Comprehensive quantitative research based on content analysis
Source: PLoS One. 2024 Mar 7;19(3):e0298601. doi: 10.1371/journal.pone.0298601 (PMC10919601; doi:10.1371/journal.pone.0298601)
Supplement: S1 Data — (ZIP) [file pone.0298601.s001.zip › Data/地级市Z_F工作报告.zip!/╦─┤¿/╥╦▒÷╩╨/└┤╫╘╣1⁄2╓┌║┼í╛┬φ┐╦╩2╛▌═°í┐_2018.docx]

各位代表：

我代表市人民政府，向大会报告工作，请予审查，并请市政协委员提出意见。

2017年工作回顾

2017年，我们在省委、省政府和市委的坚强领导下，在市人大及其常委会的法律监督、工作监督和市政协的民主监督下，深入贯彻市第五次党代会、市五届人大一次会议决策部署，稳增长、促改革、调结构、惠民生、防风险，全面完成年度各项目标任务，实现了发展速度和质量的“双提升”。

一年来，我们紧扣经济发展稳增长，发展水平迈上新台阶。主要指标好于预期。预计全市地区生产总值突破1800亿元、增长8.7%左右，增速为近5年最好水平；规模以上工业增加值增长10.3%左右；全社会固定资产投资增长15%左右；社会消费品零售总额增长13%左右；城镇居民人均可支配收入增长8.5%左右，农村居民人均可支配收入增长9.5%左右。一般公共预算收入138.81亿元、增长10.7%。发展后劲显著增强。实施重点项目227个，完成投资428.3亿元。新开工亿元以上产业项目225个，协议总投资1198.1亿元。争取到上级资金支持252.06亿元、项目1101个，其中无偿资金58.39亿元。规划储备重大项目900余个，总投资1.1万亿元，其中产业项目450个、总投资超过5000亿元。县域经济持续快速发展，翠屏区连续九年保持全省十强县称号，宜宾县进入西部百强县。园区主导产业定位全面完成，全年净增规上企业67户，总数达719户。民营经济活力持续增强，预计实现增加值1055亿元、增长9.5%，占全市GDP比重达57.8%。市场消费不断扩大。持续深化“惠民购物全川行动”“川货全国行”“万企出国门”三大活动，实现销售收入453.7亿元。成功举办首届国际（宜宾）茶业年会、中国国际名酒文化节等展会活动350余场（次），实现销售收入430.5亿元。深入实施电商示范工程，8个区县电商园区建成运营，川红茶业集团成功创建国家级电子商务示范企业，预计全市实现网络交易额220亿元、增长45%。

一年来，我们紧扣产业转型调结构，“双轮驱动”实现新突破。传统产业转型稳步推进。预计规模以上工业酒类、化工、电力等产业增加值分别增长10.5%、6.8%、12.4%；全年完成工业投资530亿元、增长33.2%，技改投资370亿元、增长12.1%。五粮液首次进入世界品牌500强。有7户企业进入全省企业100强名单，8户企业进入全省制造业企业100强名单。五粮液十万吨原酒生产、三十万吨原酒陈酿、成品酒包装及智能仓储配送一体化等50个项目开工建设，天原海丰和泰钛白粉、丝丽雅惠美线业粘胶纤维染色等40个项目快速推进，重庆嘉酿啤酒生产线技改搬迁等70个项目竣工投产。中核建中、金川电子、红星电子进入全省军民融合发展重点培育名单。预计建筑业实现总产值345亿元、增长32%，新晋升建筑施工总承包一级企业6户。新兴产业无中生有、实现重大突破。智能终端产业快速推进，规划面积8800亩的智能终端产业园，已用地4600亩，建成和在建厂房400万平方米，签约项目108个、协议总投资388.85亿元，21户企业正式投产，预计全年实现产值100亿元，全面达产后可实现年产值1900亿元。全国手机30强企业有9户落地我市。新能源汽车产业取得突破性进展，首期工程总投资45亿元、占地1000亩的汽车产业园开工建设，控股具有乘用车整车生产资质的凯翼汽车公司完成在宜宾注册和项目核准。轨道交通产业基地建设稳步推进，全面达产后可实现年产值300亿元。页岩气产业预计实现产值30.9亿元、增长90%以上，通用航空、新材料、节能环保、医药及器械、绿色食品加工业等产业发展有序推进。现代特色农业蓬勃发展。预计全市农林牧渔业总产值413亿元、增长3.5%。引进培育市级以上重点龙头企业220户，建成亿元以上农产品批发市场1个、农产品集配中心6个，创建“三品一标”农产品529个。全省农田水利现场会成功在我市召开。开工建设“宜长兴乡村振兴发展示范区”项目159个，建成酿酒专用粮基地37.1万亩。“天府龙芽”成为全省区域大品牌，我市被国际茶业委员会授予“世界著名茶乡”的称号。现代服务业活力迸发。预计全市服务业增加值增长10%，实现商品房销售面积568万平方米、增长34.3%，电信业务总量48亿元、增长50%。注册资金35亿元的四川省兴文石海天下奇文化旅游投资有限责任公司挂牌运营。蜀南竹海景区管理体制改革全面完成。创建4A级景区4个。预计实现旅游总收入540亿元、增长28.3%，接待游客5263.03万人次、增长23%。宜宾港集装箱吞吐量达38万标箱、增长26.3%。预计全市金融机构本外币存贷款余额4050亿元、增长14%。筹建创立宜宾农村商业银行，新入驻金融机构7家，金融市场秩序总体平稳。

一年来，我们紧扣重点领域促改革，创新发展开启新征程。创新驱动支撑有力。高起点规划建设占地36平方公里的大学城、科创城，与11所高校签订战略合作协议，与5所高校签订项目落地协议。四川理工·白酒学院（一期）正式建成投用，四川理工·白酒学院（二期）、电子科大宜宾研究院、西华大学宜宾研究院（一期）等项目开工建设。大力支持企业自主创新，新认定高新技术企业9户、市级工程技术研究中心2个、众创空间42个。申请专利3200件，其中发明专利810件。重点改革全面深化。深化供给侧结构性改革，“三去一降一补”各项任务全面落实。加快组建总规模200亿元的新兴产业投资基金，新兴产业公司、科教产业投资集团、城交投集团、城建集团四大国有公司挂牌成立，“1+N”国资平台模式初步形成。新开工PPP项目24个、总投资211.4亿元。国资国企改革稳步推进，五粮液薪酬制度改革、丝丽雅混合所有制改革、宜宾纸业股权划转等工作有序实施。农村资产资源确权基本完成，土地承包经营权确权通过省上验收，累计流转耕地91万亩、林地111.3万亩。率先开展“多证合一”改革，发出全省首张“二十证合一”营业执照。开放合作成效显著。预计完成招商引资市外到位资金959.21亿元、增长6.9%，其中省外到位资金583.21亿元。举办知名川商进宜宾等大型投资促进活动，共签约亿元以上产业项目240个、协议总投资1182.06亿元。设立宜宾市驻欧洲投资促进工作代表处，境外招商实现零的突破。预计外贸进出口总额达58亿元，组织企业参加境外知名专业展会、签订意向性协议4.3亿元。外事、侨务、台事、贸促、海关、检验检疫等工作取得新成绩。

一年来，我们紧扣城市发展夯基础，城市建设迈入新阶段。城市理念不断完善。按照“不求最大、但求更佳”的城市发展理念，启动新一轮城市总规和历史文化名城保护规划修编，完成城市组团控规和各类专项规划20个。按照“质量至上、精益求精”的城市建设理念，突出抓好项目设计、施工和管理，打造了一批精品工程。按照“以城建城、以城兴城”的城市经营理念，引入华侨城等企业实施区域整体开发，城市建设模式更加多元。按照“以产兴城、以城促产”的产城融合理念，统筹推进城市、园区发展，城市功能进一步完善。按照“绿色发展、和谐共享”的健康城市理念，将健康、宜居、美丽元素融入城市规划、建设、管理，得到国家卫计委等部门充分肯定。城市骨架基本形成。南部新区、岷江新区、临港新区、南溪新区建设快速推进。中心城区“两横两纵”骨干道路加快实施，金沙江公铁两用桥即将竣工、南北连接线工程开工建设，盐坪坝长江大桥快速推进，机场快速路、打营盘山隧道开工建设。长8.8公里、宽100米的金沙江大道二期、三期建设有序推进，长11.7公里、宽100米的临港长江北路改造提升工程有序实施，城市中轴线轮廓初显。南广高架建成投用，城市进出口通道形象大幅提升。县级行政区划调整稳步推进，完成乡改镇3个。城市项目快速推进。中心城区完成建设投资184亿元、增长32%。其中，三江口CBD项目一期、成贵铁路站前广场一期等项目正式开工建设，天原片区改造建设项目完成企业搬迁。城市对外通道建设加快实施，成贵铁路完成线上架梁铺轨工作；隆黄铁路加快建设；川南城际铁路（蓉昆高铁）宜宾段正式开工；成宜高速、仁沐新高速公路宜宾段开工建设，宜威高速公路起点调整方案获批。城市品质有效提升。投入15亿元，实施中心城区风貌整治工程。推动“山水园林路景”有机融合，长江生态综合治理项目丞相祠至南溪段46公里基本建成，五粮液生态湿地、金沙江湿地公园等项目开工建设。中心城区新建城市公园3个，新增绿化面积350公顷，建成区绿化覆盖率达38.2%。智慧城市、海绵城市建设有序推进。城市管理和城乡环境综合治理卓有成效，环境卫生、公共秩序、交通运行持续改善，人民群众满意度不断提高。

一年来，我们紧扣精准扶贫重实效，脱贫攻坚取得新成绩。工作基础更加扎实。实现贫困村“五个一”、贫困人口较多的588个非贫困村“三个一”全覆盖。开展贫困人口精准识别“回头看”，锁定2016年底剩余贫困人口5万户、16.84万人。扎实开展脱贫攻坚“回头帮”，0.83万“回头看”未达标贫困人口全面对标达标。脱贫攻坚档案资料统一简化。统筹推进更加有力。22个扶贫专项累计到位资金236亿元，其中财政性扶贫资金66.71亿元，完成生产和就业扶持15.07万人、移民搬迁安置3.65万人、低保政策兜底6.73万人、医疗救助7.51万人、灾后重建帮扶0.05万人。社会扶贫更加深入。1151个定点扶贫帮扶单位落实帮扶资金2.18亿元。组织2017年“扶贫日”系列活动，募集各类款项8976万元。加大援藏援彝帮扶力度，落实项目资金7230万元。考核问责更加科学。制订“1+3”脱贫攻坚考核体系，逐村逐户开展验收考评。定期进行督查巡查，常态开展暗访检查，督查暗访8轮（次），发现并整改问题788个。

一年来，我们紧扣环境保护优生态，环境质量得到新提升。污染防治“三大战役”有效落实。完成大气污染整治项目164个，中心城区空气质量二级及以上天数达261天。全面建立实施河长制，实现市县乡村四级河（段）长体系全覆盖。关闭土法造纸作坊1944个。128天建成杨湾污水处理厂主体工程。南岸污水处理厂提标改造加快推进。中心城区饮用水源水质达标率100%，“三江”宜宾段监测断面水质达标率100%。中心城区水源迁建工程加快实施。城市生活垃圾焚烧发电项目建成投用。土壤污染防治项目有序实施。节能减排任务全面完成。预计单位GDP能耗下降3.5%，公共机构单位建筑面积能耗下降3.15%。省内首家建设的福溪电厂烟气超低排放工程全面完成，9个大气污染防治攻坚项目建成投运。生态文明建设名列全省前茅。累计创建省级生态区县5个，国家级生态乡镇10个、省级73个。新建成幸福美丽新村470个，省级“四好村”120个、市级499个。生态环保工作经受住了各级的督察检验，中央环保督察组交办的326件群众来电来信按时办结率100%，省级环保督察发现的599个问题已整改557个，自然保护区完成问题整改74个，中心城区饮用水源保护区11个突出问题提前完成整改任务，几项工作均位居全省前列，圆满实现“不出重大负面事件、不出重大环保案件、不影响宜宾形象和未来发展”的工作目标。

一年来，我们紧扣民生福祉强保障，人民生活得到新改善。民生保障持续加强。全市九项民生工程、19件民生实事拨付资金112.82亿元。城镇新增就业7.33万人，城镇登记失业率3.9%。我市被确定为全省唯一的人社公共服务体系建设全域示范市。城乡居民基本医疗保险参保率达99.7%。全省基层民政工作推进会在我市召开。城乡低保和特困人员供养标准大幅提高。第三届全国“敬老文明号”和第五轮“四川省敬老模范区县”创建工作深入开展。“5·4”珙县地震、“1·28”筠连地震灾后恢复重建工作有序推进。社会事业持续发展。教育基础设施不断完善，教育质量稳步提升，全市学前教育三年毛入园率达82.9%、义务教育巩固率达94.6%、高中阶段毛入学率达88.8%。江安县顺利通过县域义务教育基本均衡国家评估认定，宜宾县等6个县顺利通过县域义务教育基本均衡省级督导评估。普通高考一本和本科上线人数连续8年增长，中职毕业生人数居全省第二。体育事业蓬勃发展，宜宾籍运动员邹敬园获世界体操锦标赛双杠冠军。卫生计生事业稳步发展，城市公立医院综合改革试点有序推进，传染病总体报告发病率连续9年处于全省最低水平。文化事业繁荣发展，成功举办“宜宾首届群众文化艺术季”等活动。翠屏区大观楼社区被评为全国15个“扫黄打非”进基层示范标兵之一。映三江农村数字电影公司被中宣部评为“服务农民、服务基层”文化建设先进集体。话剧《赵一曼》获四川省“五个一工程”优秀作品奖。王家元被表彰为全国道德模范。在全省率先实现市县整体成功创建省级文明城市。“平安宜宾”建设持续深化。立体化社会治安防控体系不断健全，“雪亮工程”和综治中心规范化建设持续推进，“创平安迎盛会”“三打击一整治”等专项行动深入开展，全市刑事案件同比下降25.4%、破案率提升15个百分点。安全生产、食品药品监管、信访维稳工作不断强化，社会保持和谐稳定。国防动员、人民防空、优抚安置工作进一步加强。司法行政、消防、气象、质量安全、应急管理、防震减灾等工作扎实推进，民族宗教、广播影视、新闻出版、电子政务、机关事务、地方志、爱国卫生、住房公积金、红十字、科协、档案、保密、工会、青少年、妇女儿童、文艺、老龄、残疾人等事业全面发展。

一年来，我们紧扣政务服务抓落实，发展环境呈现新气象。依法行政进一步强化。修订《宜宾市人民政府工作规则》，完善行政决策合法性审查机制，发挥决策咨询委员会作用，确保政府决策合法、科学、透明。健全重要文件、会议落实跟踪督查机制，先后对15项重点工作实施专项督查，行政执行更加规范、高效、务实。行政审批进一步简化。全面推进“放管服”改革，市本级保留行政审批事项219项、公共服务事项99项。开通运行网上政务服务大厅，实施“全天候”政务服务，行政审批当日办结率100%，按时办结率100%。市直部门行政审批平均承诺提速75%，为全省承诺提速率最高的市（州）。企业帮扶进一步优化。深入开展千名干部联系千户企业“进企业、结对子、解难题、促发展”等活动，共收集企业发展难题3950个，已办结2950个、办结率74.7%。认真落实各类企业扶持政策，兑现各类奖补资金3亿元。帮助企业搞好产销衔接，带领重点企业外出拓展市场10余场次。廉政建设进一步深化。常态化、制度化开展“两学一做”学习教育。严格遵守廉洁从政各项规定，进一步健全惩防体系，从源头上预防腐败。加大监察、审计力度，整肃庸政、懒政、怠政行为，全年共处置问题线索2775件，立案审查1569件，给予党纪政务处分1611人，移送司法机关30人。

各位代表，我们全力推进本届政府重点办理的十大实事，取得了明显进展。脱贫攻坚方面，圆满完成180个贫困村退出、6.75万人脱贫的年度目标任务。产业转型方面，预计传统产业增加值增长8.5%，五粮液、丝丽雅、天原主营业务收入分别增长9.9%、15%、56%；新兴产业总产值突破200亿元，智能终端、新能源汽车、轨道交通三大产业成功落地，预计通过3—5年的努力，这三大产业将实现2000亿元以上的销售收入，实现“再造一个产业宜宾”的目标。茶叶、肉牛、生猪综合产值分别达132.6亿元、155.8亿元、254.9亿元。城市建设方面，全年完成供地3.26万亩，其中中心城区供地1.56万亩、增长171.9%，预计中心城区新增建成区面积11平方公里，常住人口达119万人，城镇化率提高到48.6%。年初确定的12个重点项目完成投资83.4亿元，其中3个项目已完成，9个项目顺利推进。交通建设方面，投资101.5亿元，4条铁路、5条高速公路建设有序推进，宜宾机场迁建项目完成路基处理和航站区基础施工，宜宾港志城作业区重大件泊位完成验收。旅游建设方面，蜀南竹海、兴文石海创建5A级景区通过省级景观质量评估；李庄古镇、五粮液景区创5A级景区正抓紧完善方案。科教强市方面，新建成高等院校1所、在建3所，高等院校在校大学生达3.4万人、比上年增长27.6%。社保医保方面，“三基一卡”信息化平台建设加快推进，社会保障卡综合运用纳入全国32个试点城市范围；投资2.48亿元，率先在全省开展全民免费健康体检，按两年一轮的要求，已完成体检192.4万人。安居工程方面，投资65.6亿元，新开工危旧房棚户区改造15899套（户），其中货币化安置14294户；基本建成危旧房棚户区改造24691套（户）、公共租赁住房2792套；开工农村危房改造15561户，竣工11674户。重大水利工程方面，向家坝灌区北总干渠一期、长江上游干流四川段防洪治理工程通过国家发改委可研评估，蟠龙湖、东山、王家沟、新坝、龙滚滩、二龙滩6座中型水库建设快速推进，仁和中型水库前期工作进展顺利。生态屏障方面，大规模绿化宜宾行动扎实推进，完成营造林73.94万亩、占五年总目标任务的24.7%，新增森林面积22.26万亩、森林蓄积30万立方米。环境污染防治“三大战役”共56个重点项目，已建成投运18个。成功创建国家森林城市、国家卫生城市，在全省第一个实现中心城区和所有县城国家卫生城市（县城）全域覆盖。

各位代表，过去的一年，我们紧扣市委决策部署，先后出台一系列支持产业发展的政策措施，实施一系列推动工作的重大举措，推进一系列事关全市发展的重点工作，办成了许多打基础、利长远、可持续的重大事项。这些成绩的取得，得益于省委、省政府和市委的坚强领导，得益于市人大及其常委会和市政协的监督支持，得益于全市各级干部群众的共同努力。在此，我代表市人民政府，向各位人大代表、政协委员，并通过你们向全市各族人民、驻宜部队、武警官兵、政法干警、各民主党派、工商联、无党派人士、各人民团体和各界朋友，表示衷心的感谢，并致以崇高的敬意！

各位代表，过去一年，面对产业转型必行期、城市建设加速期、重大基础设施建设机遇期、脱贫攻坚完成期“四期”叠加形势，我们主动把握发展规律、创新发展理念、破解发展难题，取得了明显成绩、积累了宝贵经验。必须主动融入国家战略，找准宜宾定位。只有坚持以“跳出宜宾看宜宾”的勇气、“敢为天下先”的锐气、“对标先进奋争一流”的志气，切实找准全国区域中心城市、综合交通枢纽的角色定位，围绕建成“六个市”的发展目标，主动融入西部大开发、“一带一路”、长江经济带、成渝经济区等发展战略，才能拥有更加广阔的空间，实现更高层次的发展。必须深入实施双轮驱动，坚定宜宾路径。宜宾产业转型势在必行、势在快行、势在好行，只有加快走出一条传统产业持续做大做强、新兴产业强势崛起的转型发展之路，才能改变“标兵渐远、追兵渐近”的尴尬局面，夯实经济强市的核心支撑。实践证明，这条道路是完全正确的，符合经济发展规律、符合宜宾实际，必须坚定不移务实推进，决不半途而废、功亏一篑。必须始终坚持绿色发展，体现宜宾担当。只有深入践行“绿水青山就是金山银山”的理念，把“人民群众过上健康、长寿、幸福的美好生活”作为发展最终目的，切实担负起构筑长江上游生态屏障、实现宜宾永续发展的历史责任，才能最大限度得到人民群众的理解、支持和参与，形成推动发展的强大合力。必须全面深化改革创新，提升宜宾活力。只有坚持以创新的思维深化改革、扩大开放，千方百计发挥优势、深挖潜力、补齐短板，坚决破除发展进程中的一切障碍，才能抓住机遇、赢得主动、走在前列，在短短的一年内将智能终端、轨道交通打造为全省重要产业基地。必须齐心协力狠抓落实，创造宜宾速度。只要牢固树立全市“一盘棋”的理念，切实强化责任意识、协作意识、执行意识，少说不、多说行，少讲客观原因、多找主观原因，坚持当天的事情当天办，昼夜兼程、按期推进，就能创造出58天实现首批智能终端项目落地，85天完成智能终端35万平方米标准厂房及配套设施主体工程封顶建设，7个月建成四川理工·白酒学院（一期）工程，一年用地4600亩、建成和在建厂房400万平方米、签约智能终端项目108个等一个又一个“宜宾速度”。必须全力优化发展环境，打造宜宾品牌。只有始终秉持“政府与企业互利共赢、共同发展”的理念，全面优化发展政策环境、政务环境、服务环境、市场环境、人文环境，想企业所想、急企业所急，当好企业发展和项目建设的终身保姆、贴身管家，才能让企业坚定认为“选择宜宾、不会错误”、投资宜宾“前进有路、后顾无忧”，形成示范带动、比学赶超、竞相发展的良好态势。

在看到成绩的同时，我们也清醒地认识到，在决胜全面建成小康社会进程中仍然存在一些不足。传统产业转型不够深入，新兴产业发展不够充分；城市、交通基础设施建设要素保障压力大，部分项目推进不理想；县域经济发展不平衡，部分区县尚未形成特色鲜明的主导产业；脱贫攻坚、民生改善、污染防治任务艰巨；个别单位和少数干部存在不作为、慢作为、软作为等现象。此外，2017年政府工作报告分解的71项工作任务中，仍有2项没有达到目标进度。对此，我们将高度重视、认真研究、切实解决，全力确保全市经济社会平稳健康发展！

2018年工作安排

今年是贯彻党的十九大精神的开局之年、国家改革开放40周年、决胜全面建成小康社会的关键一年。新时代赋予新使命，新使命要有新作为。我们将抢抓一切机遇、付出一切努力、克服一切困难，抓重点、补短板、强支撑，奋力谱写中国梦宜宾篇章。

今年政府工作的总体要求：以习近平新时代中国特色社会主义思想为指导，深入贯彻党的十九大和中央经济工作会议精神，紧紧围绕“五位一体”总体布局和“四个全面”战略布局，全面落实省委十一届二次全会、省委经济工作暨全省金融工作会议精神和市委“565”总体谋划，坚持新发展理念，紧扣社会主要矛盾变化，按照高质量发展的要求，以人民群众对美好生活的需求为导向，以提高经济发展质量和效益为中心，以供给侧结构性改革为主线，以解决发展不平衡、不充分为着力点，以创新发展、转型发展、绿色发展为路径，推动质量变革、效率变革、动力变革，加快建设经济强市、区域中心大城市、区域交通枢纽城市、全面创新改革先行市、全面小康城市和幸福美丽城市。

今年全市经济社会发展的主要预期目标：GDP力争突破2000亿元、增长9%；规上工业增加值增长11%；建筑业总产值增长32%；服务业增加值增长10.3%；全社会固定资产投资增长13%；社会消费品零售总额增长13%；一般公共预算收入增长9%；城镇化率提高2个百分点以上；城镇新增就业7万人，城镇登记失业率控制在4.2%以内；城镇居民人均可支配收入增长8.5%；农村居民人均可支配收入增长9%；完成省上下达的物价调控、节能减排等目标任务。

今年重点办好的十件实事：

1.坚决完成年度脱贫目标任务。确保4个贫困县摘帽、167个贫困村退出、6.74万贫困人口脱贫。

2.加快产业转型步伐。传统产业增加值增长8.5%，五粮液、丝丽雅、天原主营业务收入分别达到900亿元、200亿元、175亿元；力争再有2—3个新兴产业实现突破，新兴产业总产值突破400亿元；特色农业综合产值达到895亿元。

3.推进城镇开发建设。完成城乡建设投资500亿元以上，实施中心城区20个城市重点项目、20个风貌整治项目和全市20个区域投资开发项目，中心城区新增建成区面积11平方公里、常住人口达到129万人。

4.加快重大交通项目建设。实施交通大会战，投资150亿元以上，推进4条铁路、5条高速公路建设，在建及新增铁路里程230公里以上。

5.全力打造景区品牌。力争蜀南竹海、兴文石海成功创建5A级景区，推进五粮液景区、李庄古镇创建5A级景区。

6.夯实科教强市基础。启动校（园）办学条件达标行动计划，在全省非民族市州率先实施公办普通高中学费全免，实现普及高中阶段教育。挖掘本地高校办学潜力。加快大学城、科创城建设，力争7所产研院正常运行，新增高校学生1.8万人左右。

7.启动实施乡村振兴战略。编制乡村振兴总体规划，完成100亿元规模的乡村振兴发展基金组建工作，建成产业规模3000亩以上的“乡村振兴发展示范区”11个。加强乡村服务能力建设，新建农村社区50个。

8.大力推动安居工程建设。投资35亿元，新开工危旧房棚户区改造17179套（户），基本建成保障性安居工程15281套（户）、竣工17338套（户）。实施农村人居环境整治三年行动方案，力争完成农村危房改造0.8万户、农村土坯房改造1.7万户。

9.着力抓好重大水利工程。开工建设向家坝灌区北总干渠一期、长江上游干流四川段防洪治理工程，完成中心城区水源迁建和供水保障工程建设任务，加快推进6座在建中型水库建设。

10.加快长江上游生态屏障建设。深入开展大规模绿化宜宾行动，完成营造林59.8万亩，新增森林面积22.6万亩、森林蓄积30万立方米。建成环境污染防治“三大战役”重点项目17个。完成创建国家园林城市申报工作。

一、坚持稳中求进、进中求好，进一步提升经济发展质量效益

深入实施“双轮驱动”发展战略，坚定不移做强工业、做优农业、做大服务业，加快培育一批行业领军企业，全市工业总产值突破2700亿元，三次产业结构调整为13∶54∶33。

坚定不移加快传统产业转型。优化白酒、化工、轻纺、机械、建材等产业改造升级实施方案，大力开展质量提升行动，完善配套扶持政策，继续巩固国有企业效益在全省的领先地位。抓好五粮液30万吨陶坛陈酿酒库一期、天原锂电三元正极材料及前驱体、丝丽雅雅赛尔纱新材料二期等项目建设。推动173个2000万元以上重点项目建设，确保惊雷科技2万吨双金属复合材料等82个项目竣工投产，宜宾纸业12万吨生活用纸等41个项目加快建设，长宁县竹食品产业园区等50个项目开工。推进军民融合深度发展，加快实施中核建中压水堆核燃料元件化工生产线搬迁、威力化工整体搬迁技改等项目建设。全年完成工业投资400亿元、技改投资280亿元。净增规上工业企业50户以上。继续实施建筑业大企业倍增计划，新晋升和引进建筑施工总承包一级企业8户，建筑业总产值达到448亿元。

坚定不移推动新兴产业发展。实施新兴产业发展规划，新兴产业占规上工业增加值比重达到13%。强化智能终端产业全产业链发展，确保智能云谷产业园等70个项目建成投产、实现销售收入300亿元，力争新引进企业100户以上，不断提升智能终端产业基地的影响力和辐射带动能力。加快新能源汽车产业发展，抓好宜宾凯翼汽车有限公司生产基地建设，力争实现销售收入25亿元；推进新能源汽车充电设施建设，强化汽车整车及配套零部件项目招商。加快四川轨道交通产业园建设，强化行业龙头企业引进，力争智能轨道首车下线、示范线运营。统筹推进通用航空、新材料、节能环保、医药及器械、页岩气和绿色食品加工业项目谋划和产业培育。

坚定不移发展现代特色农业。把乡村振兴作为新时代“三农”工作总抓手，健全城乡融合发展体制机制和政策体系，完善乡村振兴人才支撑、资金支持、产权制度、组织制度等保障体系。落实粮食安全主体责任，稳定粮食生产，确保粮食安全。加快建设百万亩酿酒专用粮基地。发展茶叶、肉牛、生猪、蚕桑、油樟等特色优势产业，力争综合产值分别达到160亿元、180亿元、300亿元。争创国家级出口茶叶质量安全示范市、现代农业产业园区。强化农田水利基础设施网络建设。培育发展新型经营主体200个，建设“五新”示范基地10个、“万亩地亿元钱”示范区10个，新增“三品一标”农产品50个。继续推进省级现代农业、林业、畜牧业示范县、重点县建设。

坚定不移培育壮大现代服务业。推进区域性国际商贸物流中心、会展中心建设，高水平举办迎春购物月、中国国际名酒文化节、国际茶业年会等重大活动，大力发展会展经济。推进区域性国际信息服务中心建设，推动数字经济与实体经济融合发展，积极拓展电子商务、共享经济、体验经济等消费热点。加快推进城乡物流配送网点规划建设，支持快递业加快发展。宜宾港集装箱吞吐量突破40万标箱。推进区域性国际金融中心建设，提升金融服务能力和金融业发展水平，促进金融更好服务实体经济。推进市商业银行第三轮增资扩股工作。以建设区域性国际旅游度假中心为目标，推进A级景区、旅游度假区、生态旅游示范区创建工作，大力发展乡村旅游业、康养服务业，创建一批旅游扶贫示范区、示范村和精品乡村酒店、星级农家乐，实现旅游总收入675亿元、增长25%。

坚定不移夯实县域发展支撑。实施区域协调发展战略，推进特色经济强县行动，按照“宜工则工、宜农则农、宜商则商、宜游则游”的原则，引进培育一批大型企业、骨干企业，使每个区县形成1—3个引领性、带动性、示范性强的支柱产业，加快走出一条各具特色的县域经济发展之路，推动县域经济排名稳步提升。用好用活贫困县、革命老区县、少数民族地区待遇县等扶持政策，改善县域经济发展条件。开展全民创业行动，全面实施市场准入负面清单制度，落实民营经济、中小微企业各项扶持政策，加强企业家队伍建设。进一步完善园区基础设施，健全园区准入审核机制，全年新增入驻企业150户，营业收入达到3200亿元、增长33%。

二、坚持规划引领、建管并重，进一步加快新型城镇化进程

坚持以健康城市建设为统领，不断做大城市规模、完善城市功能、提升城市品质，加快建设绿色城市、特色城市、康养城市、人文城市、美丽城市，切实践行后来居快、后来居上、后来居佳。

加快完善城市规划设计。高水平完成城市总规和历史文化名城保护规划修编，科学确定城市发展规模，进一步优化城市发展定位。推进基础教育中心、西南医疗中心等20个功能中心规划，长江大道下穿立交、中坝大桥南北桥头改造等20个城市交通节点规划，翠屏山生态保护与功能提升、白塔山生态公园打造等15个生态景观项目规划，天原片区、纸厂片区改造建设等30个精品项目规划。

加快推进城市项目建设。建立健全中心城区重大项目巡查督查、用地清理机制，及时协调解决项目建设有关问题，确保项目按既定时间节点推进。建成成贵铁路站前广场一期工程。推进三江口CBD项目建设，打造集商贸、金融、旅游、文化为一体的现代“城市客厅”。推进南部新区建设，抓紧推动金沙江大道二期、三期征地拆迁工作，确保年底13公里路基全面建成。推进天原片区改造建设，确保一季度实质性开工。推动纸厂片区改造建设。加快盐坪坝长江大桥、打营盘山隧道、金沙江公铁两用桥南北连接线工程、机场快速路、机场东连接线等项目建设。

加快提升城市整体品位。坚持在法律框架下、科学道路上、绿色生态中推进城市规划建设，提升城市舒适性、宜居性、独创性。加大城市修补力度，实施污水垃圾处理设施建设三年行动计划，加大海绵城市、综合管廊建设力度。推进“城中村”改造工作。加强城市生态修复，推进长江生态综合治理五粮液西大门至丞相祠段及盐坪坝至李庄段、五粮液生态湿地、金沙江湿地公园、七星山生态保护综合改造等项目建设，使城市与人、自然、环境和谐融合。调整优化临港开发区城市规划，加快完成城市中轴线建设，完善城市公共服务配套，提升城市管理水平，推进“产、学、研、港、城”一体化发展，打造产城融合示范区，更好地满足人民群众生产生活需要、产业发展需要、城市发展需要。

加快优化城市运营管理。加强对城市建设模式的研究和创新，做好经营土地、经营城市、经营财政“三篇文章”，多渠道筹措城市建设资金，保障项目建设顺利推进。加大城市管理力度，实施城市综合执法体制改革，强化城市管理日常巡查、监督、考核、举报机制，调动全社会参与城市管理的积极性、创造性。优化中心城区交通运行组织，进一步解决乱停乱放、占道停车等问题。完善政府购买社会服务机制，降低城市运营成本，实现常态化、长效化管理。

加快推进城乡一体化进程。扎实推进县级行政区划调整。深化户籍制度改革，推动基本公共服务向常住人口全覆盖。科学规划实施城乡“厕所革命”。结合各地资源禀赋、特色文化，培育创建一批各具特色、富有活力的生态宜居、休闲旅游、商贸物流、现代农业特色镇。全面完成4个省级幸福美丽新村示范县建设任务。建成幸福美丽新村445个，创建省级“四好村”120个、市级499个。

三、坚持对外通达、对内通畅，进一步完善立体综合交通体系

强力推进重大交通基础设施项目建设，加快构建全国性综合交通枢纽，尽快把区位优势转变为交通优势、发展优势、成果优势。

着力推进对外通道建设。建成成贵铁路宜宾段。加快推进川南城际铁路（蓉昆高铁）自宜段、隆黄铁路宜宾段建设，力争开工建设渝昆高铁、进港铁路，加快推进宜西铁路、筠盐铁路及珙叙铁路前期工作。加快成宜高速、仁沐新高速宜宾段、过境高速西段和宜彝高速公路建设进度，开工建设宜攀高速（新市至金阳段）、宜威高速公路，力争开工建设宜宾至新市高速公路，抓好南溪至内江高速公路前期工作。建成宜宾港志城作业区散货泊位，全力推动长江航道宜宾段“三升二”工程。完成宜宾机场迁建项目建设。

着力完善市域路网体系。加快推进S212宜宾至高县庆符镇公路、G353南溪至江安至泸州公路、G246筠连至巡司镇公路、兴文太平工业园区至纳黔高速江门互通公路、南溪仙源长江大桥、江安长江二桥、普和金沙江大桥等项目建设，开工建设宜叙高速竹海连接线、宜叙高速僰王山互通至兴文石海景区连接线、S215屏山至宜宾县蕨溪公路、S440长宁县下长至翠屏区公路等项目。加快5个高铁配套客运枢纽站、2个公交场站建设，建制村通客车率达到80%。强力推进“四好农村路”建设，新改建农村公路1000公里，建制村通硬化路率达到100%。

着力发挥交通枢纽功能。超前谋划高铁、港口、空港商务功能区业态发展，大力发展通道经济、港口经济。推进“交通+产业”，推动重点产业、特色农业、景区配套布局优化，实现产业全域联动、功能全域配套、服务全域覆盖。推进“交通+城镇”，推动资源、人才、资金、信息等要素向沿线城镇节点汇聚，提高城镇综合承载能力。推进“交通+民生”，加快交通连接通道和节点建设，形成客运“零距离换乘”和货运“无缝隙衔接”的交通运输网络体系，做到安全优质、高效便捷、货畅其流、人便于行。

着力健全交通服务机制。完善重大交通项目总调度、总协调机制，列出项目任务清单、问题清单、责任清单，健全要素保障和跟踪督查机制，确保每一个项目按计划实施。建立交通信息应急指挥系统，实现道路重点路段、客货运输车辆、船舶码头等动态实时监控，提高交通信息化、智慧化水平。

四、坚持创新驱动、改革推动，进一步打造内陆沿江开放高地

始终牢牢抓住创新、改革、开放三个关键，力求在重点领域和关键环节上取得新突破，增强经济社会发展动力活力。

全面推进创新驱动发展。大力实施科教兴市战略，优化大学城、科创城空间布局，完善校区、科研和商用、居住等功能配套。狠抓重点项目进度，确保四川理工·白酒学院（二期）、电子科大宜宾研究院、西华大学宜宾研究院（一期）建成并实现秋季招生入住，市科技研究中心等项目建成投用。强化产研院建设、运营和管理模式研究，加快科技成果就地转化，打造一批科技孵化器和众创空间。举办全国创新创业大赛。成功创建国家创新型试点城市，积极申报国家、省级高新区。新认定高新技术企业10户，工程技术研究中心2个。大力弘扬“工匠精神”，加快引进培育一批知识型、技能型、创新型专业人才。

全面推动重点领域改革。深化供给侧结构性改革，有效落实“三去一降一补”各项任务，加快新旧增长动能转换。推进投融资体制改革，充分发挥各类投资基金的作用，推动融资平台市场化转型。做强做优做大国有资本，改革国有资本授权经营体制，加快国有经济布局优化、结构调整、战略性重组。深化商事制度改革，持续放宽市场准入，激发各类市场主体活力。深化农村集体产权制度改革，落实农村土地“三权分置”办法，完善农村产权流转交易市场服务体系。

全面实施开放合作战略。充分用好第17届西博会、2018中外知名企业四川行等开放平台，注重分产业、分区域开展专题投资促进活动。开展委托招商、以商招商，力争全年实现新签约项目协议总投资额1200亿元以上，引进世界500强、中国500强企业5户以上。强化已签约项目跟踪对接服务，推动项目尽快落地落实。推动建立自贸试验区川南临港片区与宜宾临港开发区协同改革机制，全力申报设立宜宾临港综合保税区、国家临时开放口岸。积极参与国际贸易“单一窗口”建设，争取建设宜宾自由贸易港。加快在北美洲、香港等地设立投资促进代表处。扩大与“一带一路”沿线国家经贸交流合作，实现外贸进出口总额60亿元。

五、坚持全域统筹、精准施策，进一步加大脱贫攻坚工作力度

聚焦“两不愁、三保障”和“四个好”目标，强力推进脱贫攻坚各项工作，为全市同步全面建成小康社会再鼓干劲、再添措施、再立新功。

全力以赴落实脱贫攻坚责任。严格执行“责任在县、重点在乡、关键在村、落实到人、市县乡村分级抓落实”的领导体制和工作机制，实行挂图作战、按时推进。对照脱贫退出标准，逐村逐户逐项查短板，精准落实扶持项目，确保项目早安排、早启动、早完成。严格监管扶贫资金，全面推行公开公告公示制，接受社会监督。强化脱贫攻坚督查问责，对工作推进不力、政策落实不到位的单位和个人，实行最严厉的追责问责。

全力以赴完成年度工作目标。统筹典型示范与全面达标，统筹精准脱贫与区域攻坚，深入实施“五个一批”“六大兜底”“22个扶贫专项”年度计划。整合各类扶贫资金72亿元以上，实施基础设施、公共服务、产业发展项目，提高贫困地区经济发展整体水平。做优做强“三峡种子基金”“百企帮百村”等特色社会扶贫品牌。制订实施高县、珙县、筠连县、兴文县4个省级贫困县“摘帽”工作方案，健全领导牵头、现场推进、预评估、考核评价等工作机制，确保顺利“摘帽”。

全力以赴巩固脱贫攻坚成果。持续开展已脱贫对象“回头看”“回头帮”，对已脱贫人口、已退出村从产业就业、教育医疗、低保救助等方面给予帮扶。实施贫困家庭劳动力全域全员培训，完成劳动力实用技术培训7.6万人次，确保有劳动能力的贫困户至少有一项增收产业或有一人就业。把扶贫和扶志、扶智、开展感恩教育结合起来，大力宣传脱贫致富先进典型、先进经验，激发贫困主体发展内生动力。

六、坚持绿色优先、民生至上，进一步推动发展成果全民共享

千方百计保障和改善民生，让改革发展成果更多、更公平惠及全市人民，增强人民群众获得感、幸福感、安全感。

切实改善生态环境质量。坚决打好污染防治攻坚战，推进中央和省环保督察问题整改工作，确保按计划完成整改任务。有序推进国家环保模范城市创建工作。扎实开展环保执法专项行动，强化环境执法监管，严厉惩处污染环境、破坏生态的行为。持续实施大气、水、土壤污染防治行动计划，全面落实河长制，实施《一河一策管理保护方案》，加快城市和重点乡村生活污水、生活垃圾处理设施建设。完成区县饮用水源地环境规范化整治任务。严控主要污染物排放总量，加快淘汰落后生产能力。公共机构单位建筑面积能耗下降2.4%。统筹推进山水林田湖草系统治理，实施天然林保护二期和新一轮退耕还林工程。扎实推进省级生态县验收和生态乡镇、生态小区、生态村、生态家园创建工作。

切实推进民生工程建设。加大民生投入力度，完成省上下达的民生工程、民生实事目标任务。抓好以贫困户、高校毕业生、零就业家庭、军转复员人员和下岗再就业等为重点的群体就业，搞好农村剩余劳动力转移和返乡创业。继续抓好社保卡综合应用全国示范市和全省唯一的人民满意的人社公共服务体系全域示范市建设。完善多层次社保体系建设。继续抓好以全民免费体检为主要内容的全民预防保健工作，完善人口健康信息平台建设。推进市康复医院李庄院区整体搬迁项目。坚持“房子是用来住的，不是用来炒的”定位，加大中心城区商品住宅用地供应量，推进住房租赁制度改革，加快建立多主体供应、多渠道保障、租购并举的住房制度。推进“智慧民政”建设。加快青山殡葬园项目建设。积极发展“康养融合”服务，实施居家和社区养老服务试点，健全市县乡村四级老年教育网络，推进基层老年协会规范化建设，完成全市养老院提档升级达标。

切实统筹社会事业发展。办好优质公平教育，着力解决中小学生课外负担重、“择校热”“大班额”等问题。新建、改扩建和认定一批普惠性幼儿园。确保宜宾县、长宁县、兴文县、珙县、筠连县、屏山县6个县通过县域义务教育基本均衡国家评估认定。推动实施普通高中软实力提升工程，稳妥推进中高考改革。继续实施职业教育提品质铸品牌工程，加强校企联动，提高职业教育质量。办好民办教育、特殊教育、继续教育、民族教育和高等教育。推进市体育活动中心建设。组织参加省第十三届运动会。办好2018森林城市国际马拉松赛等活动。推进健康宜宾建设，深化公立医院改革，全面启动县域医疗卫生次中心建设。加快推进市妇幼保健院迁建、市中医院建设。开展纪念建城2200周年活动，深度挖掘本土特色文化，加强历史文化资源保护利用，提升城市品牌知名度和影响力。承办好全省第八届少数民族艺术节。加快推进市文化馆、市博物馆新馆建设。推进全国文明城市创建工作。开展第四次全国经济普查。抓好民族宗教、广播影视、新闻出版、地方志、爱国卫生、红十字、电子政务、机关事务、科协、档案、工会、青少年、妇女儿童、文艺、老龄、残疾人等事业发展。

切实创新社会治理方式。完善“党委领导、政府负责、社会协同、公众参与、法治保障”的社会治理体制，加强信息资源整合共享，提高社会治理法治化、规范化、智能化、专业化水平，打造共建共治共享的社会治理格局。健全社会治安防控体系，依法打击和惩治黄赌毒、黑拐骗等违法犯罪活动，加大网上虚假信息诈骗、倒卖个人信息等问题的打击力度。切实规范金融秩序，防范化解金融风险、企业债务风险、政府性债务风险、社保支付风险。打好禁毒人民战争。坚守社会稳定、安全生产、食品药品安全“三条底线”。构建风险管控和隐患排查“双控”预防机制，抓好高危行业领域治理和隐患排查整治。深入开展放心舒心消费城市创建工作，强化食品药品安全监管。健全灾害预警发布和公共安全体系，提升防灾减灾救灾能力。畅通信访渠道，压实信访责任，依法逐级处理群众信访诉求，确保社会稳定。

切实强化国防建设。做好国防动员、全民国防教育、人民防空、交通战备、军事设施保护、优抚安置等工作。强化国防和后备力量建设。持续支持国防和军队改革，全面落实优抚安置政策，维护军人军属合法权益，让军人成为全社会尊崇的职业。深入推进双拥模范城（县）创建工作。

全面加强政府自身建设

各位代表，为者常成，行者常至。如期实现本届政府发展目标，全面完成今年各项任务，需要我们进一步强化政治意识、大局意识、核心意识、看齐意识，不忘初心、继续前进，决不辜负党和人民重托。

尊崇法治，规范履职，切实做到依法行政。从政有法，令行为上。我们将带头弘扬社会主义法治理念、法治精神，严格依法决策、依法办事、依法履职，做到法无授权不可为、法定职责必须为。严格执行议事决策程序，自觉接受人大法律监督、工作监督和政协民主监督、社会舆论监督，着力推进政务公开和行政权力公开，让权力在阳光下运行。不断加强新型智库建设，强化决策咨询，全面提高依法行政的意识和能力，切实做到对党和人民负责、对历史负责、对自己负责。

主动作为，尽责担当，切实做到敬业勤政。勤则易成，万物皆然。我们将把事业发展放在首要位置，一门心思干工作，满怀激情干事业，挂图作战、对账销号、精准落实，绝不因个人原因影响工作。始终把工作职责记在心上、扛在肩上、抓在手上、落实到行动上，找准自己的“位”，谋好自己的“政”，干好自己的“事”，做到为人无愧于己、为政无愧于民、忠诚无愧于党，确保所做工作经得起事业发展的检验、审计的检验、历史的检验。

勤于学习，勇于创新，切实做到精谋善政。思则变，变则通，通则达。我们将坚持与时俱进、改革创新、开拓进取，切实补齐知识缺失、能力不足、本领恐慌的短板，勤于学、敏于思、精于业。始终把创新摆在发展全局的核心位置，勇于跳出思维定势，克服工作惯性，摆脱路径依赖，在创新中提升能力、破解难题，在创新中培育新动力、开创新局面。

转变作风，崇尚实干，切实做到实心实政。“口能言之，身能行之，国宝也。口不能言，身能行之，国之器也。口能言之，身不能行，国用也。口言善，身行恶，国妖也。”我们将坚持言必行、行必果、果必效、效尽佳，在一线调查研究，在一线埋头苦干，在一线解决问题。牢固树立 “功成不必在我”的理念，不计名利、不图虚功，多办顺民意、惠民生、得民心的实事，用实绩证明组织没选错人，证明群众没投错票，证明自己能干事、干好事、干成事。

雷厉风行，提升效率，切实做到高效为政。居之无倦，行之以忠。我们将按照“准、快、好”的要求，强化决策事项跟踪督导，大力整治干部不作为、慢作为、软作为等现象，提升机关行政效率。完善干部考核评价机制，建立激励机制和容错纠错机制，旗帜鲜明为那些敢于担当、善于执行、踏实做事、不谋私利的干部撑腰鼓劲，形成干事创业的良好氛围。

严于律己，从严执纪，切实做到廉洁从政。源清则流清，心正则事正。我们将严格落实党风廉政建设责任制，认真贯彻中央八项规定实施细则精神、省委省政府十项规定实施细则要求和市委市政府十项规定，大力倡俭治奢，坚决纠正“四风”。加大过程监管和审计监督力度，着力构建亲清新型政商关系，依法依规、理直气壮服务市场主体。深入推进“两学一做”学习教育常态化制度化。扎实开展“不忘初心、牢记使命”主题教育。始终保持反腐败高压态势，努力营造风清气正的政治生态和社会环境。

各位代表，积力所举，无往不胜；众智所为，无所不成。站在新起点、实现新跨越，是时代进步的必然要求、经济发展的现实需要、全市人民的共同期盼，更是我们的神圣职责。让我们更加紧密地团结在以习近平同志为核心的党中央周围，在省委、省政府和市委的坚强领导下，在市人大及其常委会和市政协的监督支持下，紧紧依靠全市人民，紧盯目标不动摇、奋力攻坚不松劲、狠抓落实不懈怠，只争朝夕、锐意进取、埋头苦干，为决胜全面建成小康社会而努力奋斗！
